# Principles and Practice of Head and Neck Oncology

**DOI:** 10.1038/sj.bjc.6601592

**Published:** 2004-01-20

**Authors:** R S Weber, K B Pytynia

**Affiliations:** 1 MD Anderson Cancer Center, Department of Head and Neck Surgery, 1515 Holcombe Blvd, Unit 441, Houston, TX 77030, USA

The editors aim to deliver a comprehensive text on malignancies affecting the head and neck, which is both beneficial to experienced surgical, radiation and medical oncologists and yet an easily understandable introductory text for those less familiar with head and neck oncology. This text attempts the overwhelming task of describing the various types of carcinoma of the head and neck, their epidemiology, treatment and outcomes. This text is unique in that it accurately describes both the surgical and radiotherapy treatments available for various types of head and neck cancer, though in future editions the editors may wish to include more information regarding the emerging use of chemotherapy as an adjunctive treatment. Each chapter includes practical clinical advice and treatment algorithms useful for those who do not regularly deal with patients who have cancer of the head and neck. This multidisciplinary approach makes the ‘Principles and Practice of Head and Neck Oncology’ an excellent reference for those who have head and neck oncology patients as part of their medical or surgical practice.

The introduction includes a well-researched but under-referenced discussion of epidemiology of head and neck cancer. The molecular biology chapter is understandably an introduction to the complex field of cancer biology, including common techniques and the current theories on progression of dysplasia of head and neck cancers, and even goes so far as to include a glossary of common molecular biology terms useful for those who do not regularly deal with basic science research. Imaging in head and neck cancer contains a well-thought-out comparison of the various imaging techniques used today, and contains a number of examples that may guide the practitioner in determining the appropriate imaging for a particular tumour. The discussions of the principles of radiotherapy and chemotherapy are excellent reviews, although the toxicity associated with chemotherapy and the current status of clinical trials involving chemotherapy could be further explored. The anaesthesia chapter highlights the difficulties in providing anaesthesia for the head and neck cancer patient, and the necessity for communication between the surgeon and anaesthesia provider. The chapters on nursing care, nutrition, dental management and palliation further support the necessity of the multidisciplinary approach for the head and neck cancer patient, along with providing practical reviews.

As the majority of head and neck cancers are squamous cell carcinomas, the first half of the text concentrates on the diagnosis and management of squamous cell carcinomas. The chapters are divided according to the major subsites of squamous cell carcinoma of the head and neck, specifically oral cavity, oropharynx, hypopharynx and larynx. Each chapter begins with a discussion of anatomy and is well illustrated, with pictures of initial lesions, surgical photographs and outcomes. Treatment discussions also include functional results associated with each modality. The chapters vary in depth in which each subsite is covered, in that some chapters cover specific surgical techniques while others include radiation treatment planning. Each chapter contains a well-thought-out schema of treatment algorithms and detailed diagrams of staging, often including multiple staging systems. The authors include complete references of published studies, but the discussion of the referenced studies could be summarised into a cohesive treatment paradigm for some topics. The management of the neck chapter provides a thorough history of the evolution of the treatment of neck disease.

The remaining chapters in the book focus on tumours encountered more infrequently, including those involving the paranasal sinuses, skull base, parapharyngeal space and salivary glands. The discussions of thyroid cancer, melanoma and free flaps are thorough and practical.

In conclusion, ‘The Principles and Practice of Head and Neck Oncology’ provides the reader with a practical review of head and neck oncology. The need for a multidisciplinary approach and the continued use of organised clinical trials is emphasised throughout the text. This text will be a resource for residents and those pursuing advanced training in head and neck oncology. For the practitioner, ample references are provided for more in-depth review as well as insight into the contributor's treatment philosophy.

